# Structural and Mechanistic Characterization of *Mycobacterium tuberculosis* TrxR Inhibition by Glutathione-Coated Gold Nanocluster

**DOI:** 10.3390/ijms27031209

**Published:** 2026-01-25

**Authors:** Zhaoyang Li, Wenchao Niu, Dongfang Xia, Yuanyuan Chen, Sixu Chen, Botao Zhang, Junshuai Wang, Haojia Zhu, Huai Yang, Fei Xie, Yubai Zhou, Yong Gong, Yuancong Xu, Peng Cao

**Affiliations:** 1 Beijing Key Laboratory of Cardiopulmonary-Cerebral Resuscitation Innovation and Translation, College of Chemistry and Life Science, Beijing University of Technology, Beijing 100124, China; zhaoyangli@emails.bjut.edu.cn (Z.L.); xiadf@ihep.ac.cn (D.X.); sixuchen@emails.bjut.edu.cn (S.C.); zhbbt@emails.bjut.edu.cn (B.Z.); wjunshuai@emails.bjut.edu.cn (J.W.); zhj2023@emails.bjut.edu.cn (H.Z.); yanghuai@emails.bjut.edu.cn (H.Y.); xiefei990815@bjut.edu.cn (F.X.); zhouyubai@bjut.edu.cn (Y.Z.); 2 Beijing Synchrotron Radiation Facility, Institute of High Energy Physics, Chinese Academy of Sciences, Beijing 100049, China; wcniu@ihep.ac.cn (W.N.); yonggong@ihep.ac.cn (Y.G.); 3 The Research Platform for Protein Sciences, Institute of Biophysics, Chinese Academy of Sciences, 15 Datun Road, Chaoyang District, Beijing 100101, China; chenyy@ibp.ac.cn; 4 Institute of Matter Science, Beijing University of Technology, Beijing 100124, China

**Keywords:** thioredoxin reductase, *Mycobacterium tuberculosis*, gold nanocluster, X-ray crystallography, molecular dynamics simulations

## Abstract

*Mycobacterium tuberculosis* (*M. tuberculosis*) relies on the thioredoxin (Trx)–thioredoxin reductase (TrxR) system to maintain intracellular redox homeostasis and to support Trx-dependent DNA synthesis and repair, making TrxR a potential target for anti-tuberculosis therapy. Gold nanoclusters have been reported to inhibit human TrxR and suppress tumor growth, suggesting that gold-based nanomaterials can modulate TrxR activity. In this study, we report a previously uncharacterized oxidized crystal structure of *M. tuberculosis* TrxR containing two dimers in the asymmetric unit and use this structure to investigate inhibition by a glutathione-coated gold nanocluster (GSH-AuNC). Biolayer interferometry and enzymatic assays show that GSH-AuNC binds directly to *M. tuberculosis* TrxR and efficiently inhibits its catalytic activity at the purified enzyme level. Molecular dynamics simulations indicate that GSH-AuNC can occupy a surface pocket proximal to the active site, providing a plausible structural basis for enzyme engagement. AlphaFold3 modeling of the *M. tuberculosis* TrxR-Trx heterodimeric complex defines the interaction interface required for productive electron transfer and provides a structural hypothesis for how GSH-AuNC disrupts this process. Together, these results provide structural and mechanistic insights into the biochemical modulation of *M. tuberculosis* TrxR by GSH-AuNC, while the antimycobacterial activity of GSH-AuNC remains to be evaluated in future studies.

## 1. Introduction

*Mycobacterium tuberculosis* (*M. tuberculosis*) is the primary pathogen causing tuberculosis (TB) and has long posed a threat to global public health [1]. In 2023, approximately 10.8 million people worldwide contracted TB, with about 1.25 million deaths [2]. Although TB is generally curable with timely and appropriate treatment, delayed diagnosis, limited access to healthcare, and the increasing prevalence of drug-resistant strains continue to drive its global mortality burden. Despite substantial progress in controlling the spread of *M. tuberculosis* and treating patients with anti-TB drugs, the rise of drug-resistant strains has significantly reduced the efficacy of traditional medications [3,4,5]. This underscores the urgent need to develop new anti-TB strategies and accelerate the discovery of new therapeutics to address the growing burden of drug resistance.

The thioredoxin (Trx)–thioredoxin reductase (TrxR) system plays a critical role in the survival and virulence of *M. tuberculosis*, making it an attractive drug target [6]. TrxR is a nicotinamide adenine dinucleotide phosphate (NADPH)-dependent enzyme containing a flavin adenine dinucleotide (FAD) domain and belongs to the family of pyridine nucleotide–disulfide oxidoreductase flavoproteins. Together with Trx and NADPH, TrxR forms the Trx-TrxR system, which is essential for maintaining intracellular redox homeostasis [7]. Unlike many other organisms, including humans, *M. tuberculosis* lacks the glutathione system, which is another critical redox-balancing system composed of glutathione, glutaredoxin, glutathione reductase, and NADPH [8,9]. This system not only maintains redox balance but also plays a vital role in antioxidant defense, regulation of metabolic pathways, and protection against reactive oxygen species (ROS)-mediated damage [10]. Moreover, the Trx-TrxR system supports nucleic acid integrity, particularly by activating ribonucleotide reductase during DNA replication and repair, where it is crucial for repairing oxidative damage [11,12].

Over the past decade, gold nanoclusters (AuNCs) have demonstrated significant potential in a wide range of biomedical applications [13,14,15,16]. AuNCs are characterized by an ultrasmall size, good biocompatibility, favorable safety profiles, and reported broad-spectrum anti-inflammatory activity [17]. Beyond these general advantages, AuNCs can modulate redox-active proteins by releasing Au(I) ions that preferentially react with thiol groups. Gold(I) complexes (e.g., auranofin) and AuNCs have been reported to inhibit overexpressed TrxR in human cancer cells, while exhibiting limited toxicity toward normal cells [18,19,20,21]. Notably, a Cyclo(-RGDfK)-YCC peptide-coated AuNC was shown to bind human thioredoxin reductase (hTrxR), triggering ROS-mediated oxidative damage and suppressing tumor growth [18,19]. Consistently, glutathione-coated AuNC (GSH-AuNC) was reported to inhibit the SARS-CoV-2 main protease (M^pro^) in both cellular and animal models by targeting the catalytic cysteine residue [22]. AuNCs have also been explored therapeutically in other diseases. For example, R-dihydrolipoic acid (R-DHLA)-stabilized cerium-modified AuNCs (R-DHLA-AuNCs-Ce) were developed for the treatment of advanced rheumatoid arthritis [23]. Moreover, their versatile surface functionalization further makes AuNCs attractive candidates for nucleic acid delivery systems, offering additional opportunities to expand and enhance treatment strategies [24].

Motivated by the ability of AuNCs to modulate hTrxR, we investigated whether GSH-AuNC could target *M. tuberculosis* TrxR (UniProt: P9WHH1, 335 amino acids) and sought to characterize the structural and mechanistic basis of this inhibition. In this study, we first determined a previously unreported crystal form of *M. tuberculosis* TrxR by X-ray diffraction, and then examined the interaction between GSH-AuNC and TrxR, along with its inhibitory potency, using biolayer interferometry (BLI) and enzymatic assays. To interpret these observations at the molecular level, we combined molecular dynamics (MD) simulations with AlphaFold3-based modeling of the *M. tuberculosis* TrxR-Trx heterodimeric complex to probe electron-transfer geometry and the potential mechanism of inhibition by GSH-AuNC.

## 2. Results

### 2.1. Protein Preparation and Crystal Growth

**Figure 1:**
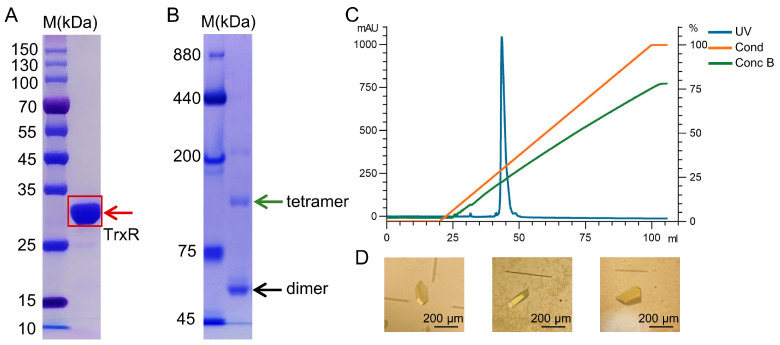
Purification and crystallization of *M. tuberculosis* TrxR. (**A**) SDS-PAGE analysis of purified TrxR. (**B**) Blue native PAGE analysis of TrxR. The positions of the tetramer and dimer are indicated by green and black arrows, respectively. (**C**) Elution profile from anion-exchange chromatography. (**D**) Representative crystals.

To obtain recombinant protein in *Escherichia coli* (*E. coli*), we expressed the functional domain of *M. tuberculosis* TrxR (residues 14–321), yielding soluble protein. The protein was purified by Ni-NTA affinity and anion-exchange chromatography, with FAD supplemented during purification to promote cofactor incorporation, yielding a highly purified holoenzyme. SDS-PAGE showed a predominant band at ~33 kDa, consistent with the expected molecular mass of *M. tuberculosis* TrxR (Figure 1A). Blue native PAGE (Figure 1B) and analytical ultracentrifugation (Supplementary Figure S1) indicated that TrxR predominantly exists as a dimer in solution, with a minor population corresponding to tetramers. Anion-exchange chromatography produced a single sharp peak, supporting the high purity of the preparation for crystallization (Figure 1C). TrxR crystals were obtained using the sitting-drop vapor-diffusion method, and their yellow color was consistent with FAD incorporation (Figure 1D).

### 2.2. Crystal Structure Analysis of M. tuberculosis TrxR

**Figure 2:**
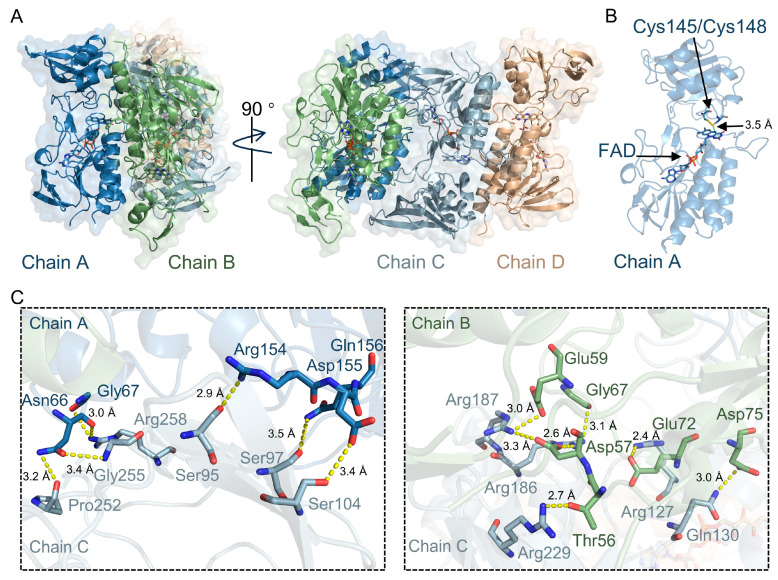
Crystal structure of *M. tuberculosis* TrxR. (**A**) Overall structure of TrxR containing four molecules in the asymmetric unit, with the FAD cofactors depicted as sticks. (**B**) Structure of one TrxR monomer. The active-site cysteines are shown as sticks. The distance between FAD and the active-site disulfide is indicated by a green dashed line. (**C**) Detailed view of interactions at the interfaces between TrxR dimers in the asymmetric unit. Interfacial residues are shown as sticks, and hydrogen-bond interactions are indicated by yellow dotted lines.

The crystal structure of *M. tuberculosis* TrxR was determined by X-ray diffraction at a resolution of 2.6 Å (Figure 2A). Unlike the reported *M. tuberculosis* TrxR structure deposited in the Protein Data Bank (PDB code: 2A87, space group *P*4_1_2_1_2) [25], our crystal represents a previously unreported crystal form. It crystallized in space group *C*222_1_ and contains four molecules arranged as two dimers in the asymmetric unit (Supplementary Table S1). Each monomer contains FAD- and NADPH-binding domains, and the overall arrangement corresponds to an oxidized FO (FAD-oxidizing) conformation, in which the active-site disulfide is positioned proximal to FAD (Figure 2B). FAD is clearly defined in the electron density map (Supplementary Figure S2). No electron density for NADPH was observed in the NADPH-binding domain. Analysis of the electron density map revealed that most residues in chains A and C exhibited well-defined conformations and clear density signals. In contrast, residues 186–200 in the NADPH-binding domain of chain D, as well as residues 133–157 and 186–243 in the corresponding domain of chain B, showed poor electron density. This local disorder is consistent with the conformational plasticity of the NADPH-binding domain required for NADPH engagement and catalysis [25].

We used PISA (https://www.ebi.ac.uk/pdbe/pisa/, accessed on 18 December 2025) to analyze the oligomeric state of our structure. The results reveal that within the asymmetric unit, chains A/B and C/D form a canonical dimer (buried surface area ≈ 2264–2500 Å^2^, Δ*G*_int_ ≈ −7 to −9 kcal/mol). These two dimers further associate through a polar interface (~650 Å^2^) without the involvement of crystal lattice symmetry. Superposition of the two dimers yielded a C*α* root-mean-square deviation (RMSD) of 0.31 Å, indicating near-identical structures. The dimer–dimer interface is mediated by the residues Ser95, Ser97, Ser104, Pro252, Gly255, Arg258, Arg127, Gln130, Arg186, Arg187, and Arg229 from chain C, which form contacts with the residues Arg154, Gln156, Asp155, Asn66, and Gly67 from chain A, as well as Glu72, Asp75, Asp57, Gly67, Glu59, and Thr56 from chain B (Figure 2C).

To further assess structural differences, we compared our structure with previously published TrxR structures [25,26,27,28]. The RMSD between the monomer of our structure and the published *M. tuberculosis* TrxR structure (PDB code: 2A87) [25] was approximately 0.38 Å, indicating a high degree of structural similarity in the monomeric region (Supplementary Figure S3). Interestingly, sequence and structural comparisons of our TrxR monomer with those of *M. tuberculosis* (PDB code: 2A87) [25], *Mycobacterium smegmatis* (*M. smegmatis*) (PDB code: 8CCI) [26], *E. coli* (PDB code: 1TDE) [27], *Haemophilus influenzae* (*H. influenzae*) (PDB code: 5U63), and *Helicobacter pylori* (*H. pylori*) (PDB code: 2Q0L) [28] reveal a conserved CXXC motif, along with FAD-binding and NADPH-binding domains (Supplementary Figure S4A,B). Despite these conserved regions, overlay analysis of TrxR structures from other species showed RMSD values ranging from 0.67 to 6.83 Å (Supplementary Table S2), indicating varying degrees of conformational flexibility in the NADPH-binding domains. Notably, the NADPH-binding domain in *M. tuberculosis* shows a larger deviation relative to that in *H. influenzae* (Supplementary Figure S4C).

### 2.3. Analysis of the Direct Interaction Between GSH-AuNC and TrxR

A previous study reported that AuNCs can directly target and inhibit hTrxR [18]. Building on these findings, we next investigated whether GSH-AuNC also inhibits *M. tuberculosis* TrxR directly. GSH-AuNC was synthesized according to previously reported protocols [22,29], and its physicochemical properties were characterized, showing good agreement with published data (Supplementary Figure S5). In addition, cell viability assays in human bronchial epithelial 16HBE cells did not reveal obvious cytotoxicity under the tested conditions (Supplementary Figure S6A).

**Figure 3:**
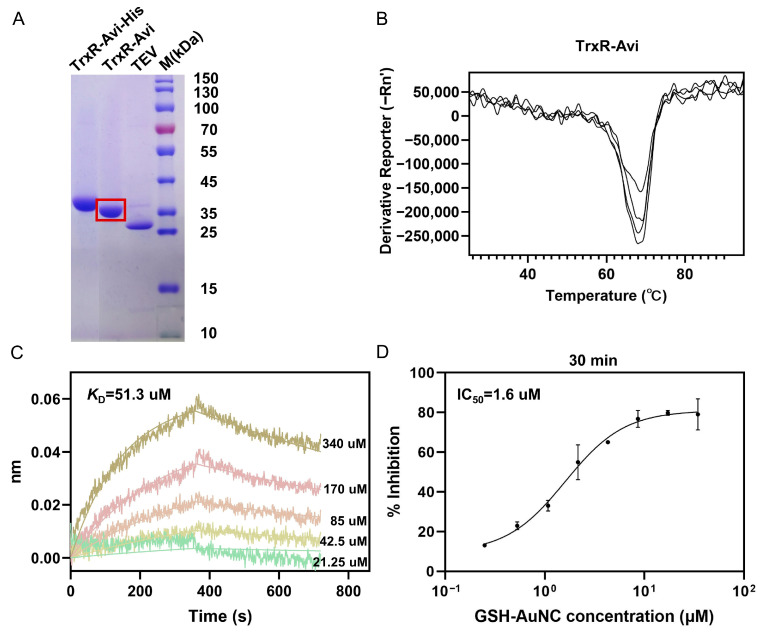
Interaction of GSH-AuNC with *M. tuberculosis* TrxR and inhibition of TrxR activity. (**A**) SDS-PAGE analysis of purified *in vivo*-biotinylated TrxR (TrxR-Avi). Lanes 1–3 show TrxR-Avi-His, TrxR-Avi, and TEV protease used for His-tag cleavage. The red box indicates the biotinylated TrxR used for BLI. (**B**) Thermal shift assay of TrxR-Avi. (**C**) BLI sensorgrams showing binding of GSH-AuNC to TrxR (*K*_D_ determined by global fitting). (**D**) Inhibition of TrxR activity by GSH-AuNC. Data are presented as mean ± SD from three biological replicates.

We first introduced an AviTag at the C-terminus of recombinant *M. tuberculosis* TrxR, thereby enabling in vivo biotinylation during protein expression (Figure 3A). To assess whether the addition of this tag affected the protein’s folding or structural integrity, we evaluated the biotinylated TrxR (TrxR-Avi) using a thermal shift assay (Figure 3B). The results indicated that TrxR-Avi remained thermally stable, supporting its use as the immobilized protein on the biosensor in subsequent BLI binding assays.

Subsequently, we measured GSH-AuNC and TrxR binding using BLI. TrxR-Avi was immobilized onto the surface of the SA biosensor through the high-affinity biotin-streptavidin system. Once the protein was immobilized, the sensor was sequentially immersed in a series of GSH-AuNC solutions at varying concentrations for binding reactions, then transferred to a running buffer alone for dissociation reactions. BLI data analysis showed concentration-dependent responses for GSH-AuNC binding to TrxR. Global fitting of the binding–dissociation kinetic data yielded an equilibrium dissociation constant (*K*_D_) of 51.3 μM (R^2^ = 0.978) (Figure 3C). These data confirm a direct interaction between GSH-AuNC and TrxR.

### 2.4. Inhibition of TrxR Activity by GSH-AuNC

To evaluate the inhibitory effect of GSH-AuNC on *M. tuberculosis* TrxR, we measured the TrxR catalytic activity using the DTNB reduction assay. TrxR was incubated with GSH-AuNC over a range of concentrations prepared by two-fold serial dilution, and activity was monitored by measuring absorbance at 412 nm with a microplate reader. GSH-AuNC inhibited TrxR in a concentration-dependent manner. Dose–response fitting yielded an IC_50_ of approximately 1.6 µM with a 95% confidence interval of 1.1–2.1 µM, with the goodness of fit indicated by R^2^ = 0.974 (Figure 3D). Inhibition increased with increasing GSH-AuNC concentration, reaching ~82% inhibition at 35 µM. Extending the pre-incubation time from 10 to 60 min at 37 °C, during which TrxR was pre-incubated with GSH-AuNC prior to initiating the reaction, decreased the apparent IC_50_ from 2.1 to 1.1 μM (Supplementary Figure S6B). Together, these results show that GSH-AuNC efficiently inhibits *M. tuberculosis* TrxR.

### 2.5. Molecular Dynamics Simulations of the Interaction Between GSH-AuNC and TrxR

We performed MD simulations to model the binding of GSH-AuNC to *M. tuberculosis* TrxR to gain insight into potential binding modes by which GSH-AuNC inhibits TrxR activity. The GSH-AuNC structure, Au_29_(GSH)_27_, was built according to the previously reported model [30]. It comprises a gold core coordinated by glutathione ligands through Au-S bonds and was subsequently geometry-optimized using quantum-chemical simulation software (Supplementary Figure S5F). Based on the GSH-AuNC structure, MD simulations were performed using the NAMD software package, and a 100 ns trajectory was generated.

**Figure 4:**
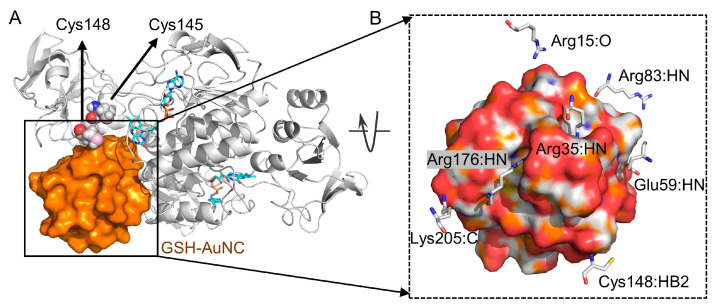
MD simulation analysis of the interaction between GSH-AuNC and *M. tuberculosis* TrxR. (**A**) The binding mode between GSH-AuNC and TrxR. GSH-AuNC (in surface) and TrxR (in cartoons) are colored in orange and gray, respectively. The FAD cofactors are shown as sticks. Two active-site cysteine residues are shown as spheres. (**B**) Close-up view of the interaction between GSH-AuNC and TrxR. Residues at the binding interface are shown as sticks and labeled.

The RMSD of the protein backbone was monitored to evaluate system stability (Supplementary Figure S7A). The RMSD results showed that the system stabilized at around 5 ns and remained stable until the end. The average conformation extracted from the 50–100 ns interval of the dynamic trajectory revealed that GSH-AuNC bound stably near the hydrophobic cavity between the two monomers (Figure 4A). We further analyzed the interactions between GSH-AuNC and TrxR. Hydrogen bonds were observed between GSH-AuNC and the residues including Arg35, Glu59, Arg83, Cys148, Arg176, and Lys205, while salt bridges were formed with Arg83 and Lys205, as shown in Figure 4B. Molecular mechanics/Poisson-Boltzmann surface area (MM/PBSA) was used to estimate the Gibbs free binding energy between GSH-AuNC and TrxR. We uniformly extracted 20 conformations from the molecular dynamics trajectory for MM/PBSA calculations. The average Gibbs free binding energy (Δ*G*) for the trajectory in the 50–100 ns range was −5.24 kcal/mol (Supplementary Figure S7B), which is comparable to the experimental value. The Δ*G*_exp_ calculated from the experimental *K_D_* was −5.89 kcal/mol (Δ*G* = *RT* ln(*K_D_*), *T* = 300 K, *K_D_* = 51.3 μM).

### 2.6. AlphaFold3 Modeling Suggests an Electron-Transfer Process Between TrxR and Trx

**Figure 5:**
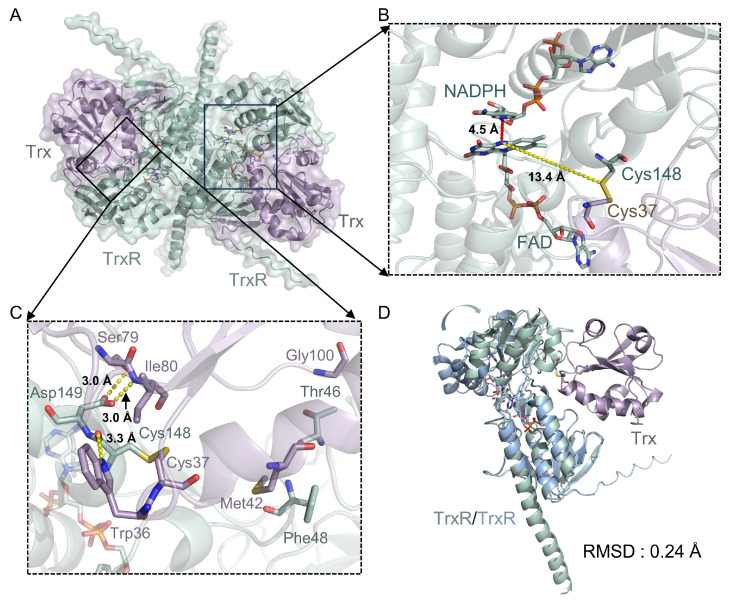
AlphaFold3 modeling of the *M. tuberculosis* TrxR-Trx complex. (**A**) Predicted TrxR-Trx heterodimeric complex with the key active-site residues and NADPH and FAD cofactors displayed as sticks. Predicted TrxR and Trx are colored in green and purple. (**B**) Close-up view of the NADPH-FAD and FAD–disulfide distances. The distance between FAD and NADPH is indicated by red dashed lines, while the distance between FAD and the active-site disulfide is indicated by yellow dashed lines. (**C**) Detailed view of the interaction interface between TrxR and Trx. The interacting residues are shown as sticks. Hydrogen-bond interactions are indicated by yellow dotted lines. (**D**) Superposition of the predicted complex model with our structure, aligned based on the FAD-binding domain. Our TrxR is colored in blue.

To assess the interaction and electron-transfer mechanisms, we employed AlphaFold3 to predict the heterodimeric TrxR-Trx complex of *M. tuberculosis* (Figure 5A–C and Supplementary Figure S8A). NADPH and FAD were included in the prediction to simulate the catalytically relevant reduced state. The predicted TrxR-Trx model was superposed with the crystal structure of Trx (PDB code: 2I1U) [31] and our TrxR structure. The RMSD values for Trx and TrxR were 0.26 Å and 6.71 Å, respectively (Supplementary Figure S8B,C). In parallel, *M. tuberculosis* TrxR was independently predicted by AlphaFold3 and compared with our crystal structure, yielding a low RMSD of 0.39 Å (Supplementary Figure S8D). These results indicate that the larger deviation observed for TrxR in the TrxR-Trx complex arises from a substantial conformational rearrangement that occurs upon Trx binding. Specifically, the FAD-binding domain aligns closely with the experimental structure (RMSD 0.24 Å), whereas the NADPH-binding domain undergoes a pronounced swinging motion toward Trx (Figure 5D). This behavior is similar to the conformational change reported for the structure of *E. coli* TrxR-Trx complex (PDB code: 1F6M) [32]. Superposition of the *M. tuberculosis* TrxR-Trx model with the *E. coli* TrxR-Trx crystal structure yielded an overall RMSD of 1.19 Å, indicating a similar relative positioning of TrxR and Trx in the two complexes (Supplementary Figure S8E).

In the predicted model, hydrogen-bond interactions at the TrxR-Trx interface were mediated by Asp149 in TrxR, and Ser79, Ile80, and Trp36 in Trx (Figure 5C). Furthermore, distance analysis shows that the nicotinamide ring of NADPH is positioned approximately 4.5 Å from the flavin ring of FAD, while the distance between FAD and the active-site disulfide bond is 13.4 Å (Figure 5B). This configuration stands in contrast to the FO conformation, in which the active-site disulfide is proximal to FAD (PDB code: 2A87) [25], where the NADPH-FAD and FAD–disulfide distances are 16.4 Å and 3.7 Å, respectively (Supplementary Figure S3B). These distances are consistent with an FR-like (FAD-reducing) conformation, similar to that observed in the *E. coli* TrxR-Trx complex [32]. Overall, the AlphaFold3 model suggests that, in this conformation, the NADPH-binding domain undergoes a substantial rotation that could facilitate electron transfer from NADPH to FAD in bacterial TrxR-Trx complexes.

## 3. Discussion

In this work, we present a structural analysis of *M. tuberculosis* TrxR and explore its modulation by GSH-AuNC. The structural, biophysical, and computational data together suggest that the TrxR-Trx system is conformationally flexible and may be susceptible to interference by GSH-AuNC in the catalytic region. However, this study is limited to purified-enzyme and structural analyses, and the antimycobacterial efficacy of GSH-AuNC remains to be evaluated in cellular models.

In addition to the canonical TrxR dimer [25], our crystal form reveals a dimer–dimer association mediated by a relatively small polar interface. Consistent with this crystallographic observation, blue native PAGE and analytical ultracentrifugation indicate that TrxR exists predominantly as a dimer, with a minor population consistent with a tetrameric species. Given the limited interface area, this higher-order assembly may reflect crystal packing or a low-abundance state in equilibrium with dimers. Elevated protein concentrations or oxidative conditions characteristic of the mycobacterial phagosome could potentially shift this equilibrium toward the tetrameric state. Meanwhile, the dimer–dimer interface overlaps with the putative Trx-binding surface, implying that the tetrameric arrangement would likely be incompatible with productive Trx engagement. However, the physiological relevance and functional significance of the TrxR tetramer remain to be determined.

**Figure 6:**
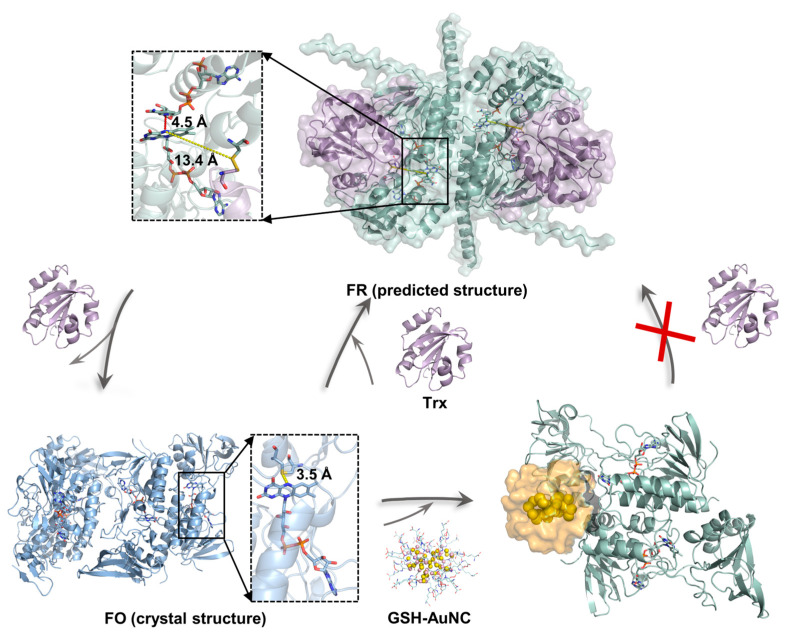
Proposed catalytic cycle and inhibition mechanism of *M. tuberculosis* TrxR. The catalytic cycle involves a transition from the FR conformation (**top center**, modeled by AlphaFold3) to the FO conformation (**bottom left**, our structure). In the FR conformation, the distances between NADPH-FAD and FAD–disulfide are 4.5 Å and 13.4 Å, respectively. In the FO conformation, the distance of FAD–disulfide is 3.5 Å. GSH-AuNC is proposed to bind a surface pocket proximal to the TrxR active site, potentially perturbing TrxR-Trx coupling and electron transfer. Predicted TrxR and Trx are colored in green and purple, and our TrxR is colored in blue. GSH-AuNC is colored in orange. The red cross indicates that Trx binding is blocked.

A schematic of the proposed catalytic cycle of *M. tuberculosis* TrxR and the proposed inhibition mode of GSH-AuNC are shown in Figure 6. Our crystal structure captures TrxR in an oxidized FO conformation and reveals substantial disorder in the NADPH-binding domain, as indicated by weak electron density, consistent with other oxidized TrxR structures [25,26,27,28]. This disorder likely reflects the conformational plasticity required for catalytic turnover. We further used AlphaFold3 to model the *M. tuberculosis* TrxR-Trx heterodimer in an FR-like state (Figure 5). The predicted complex is compatible with the “swing-and-exchange” paradigm described for *E. coli* TrxR [32], in which the NADPH-binding domain rotates to bring the nicotinamide ring of NADPH into proximity to the FAD isoalloxazine ring. Such a rearrangement could enable hydride transfer from NADPH to FAD and subsequent reduction of the active-site disulfide. Accordingly, overlay of our crystal structure with the predicted FR-like model highlights a pronounced domain rearrangement between the FO and FR states (Figure 6).

MD simulations indicate that GSH-AuNC can associate with a surface pocket adjacent to the active-site region of *M. tuberculosis* TrxR. The nanocluster remains stably positioned in this pocket and forms multiple polar and electrostatic contacts with residues surrounding the catalytic center (Figure 4), consistent with the direct binding and inhibition observed experimentally. Notably, the predicted GSH-AuNC binding site partially overlaps with the putative Trx-binding surface (Supplementary Figure S7C), suggesting that nanocluster engagement could impede Trx association and thereby compromise subsequent electron transfer (Figure 6). Moreover, in the enzyme inhibition assay, extending the pre-incubation time to 60 min decreased the apparent IC_50_, which may be consistent with a conformational adjustment of TrxR upon nanocluster binding. As another possibility, previous studies have reported that gold complexes can release Au(I) ions that coordinate cysteine thiols in target proteins [18,22,29]. On this basis, we hypothesize that Au(I) derived from GSH-AuNC could coordinate the active-site cysteines (Cys148 and Cys145) and thereby inhibit TrxR activity. To distinguish between these possibilities, competition assays involving Trx and GSH-AuNC, together with mass spectrometry analyses to probe Au(I)-cysteine adduct formation, could be employed. In addition, potential off-target modification of other cysteine-containing cellular proteins cannot be excluded. These proposed models and mechanisms remain to be tested and will require further experimental validation.

Prokaryotic TrxRs are low-molecular-weight enzymes, whereas eukaryotic TrxRs are high-molecular-weight proteins [33,34], and our sequence and structural comparison highlights substantial architectural divergence between *M. tuberculosis* TrxR and hTrxR (PDB code: 7X1R; Supplementary Figure S9). From a biological perspective, *M. tuberculosis* lacks a glutathione-based redox system and is therefore highly dependent on the Trx-TrxR pathway for intracellular redox homeostasis [8]. By contrast, mammalian cells possess additional glutathione-dependent pathways that could partially buffer redox perturbations [35]. Together, these features suggest that TrxR may be a potential anti-TB target. Future studies should evaluate the antimicrobial and bactericidal efficacy of GSH-AuNC against *M. tuberculosis* in relevant infection models. To improve selectivity, AuNCs conjugated with targeted peptides or nucleic acids could be engineered to enhance binding to the *M. tuberculosis* TrxR interface, potentially increasing both affinity and specificity.

In summary, by integrating X-ray crystallography, BLI, an enzymatic inhibition assay, MD simulations, and AlphaFold3 modeling, we provide the structural basis and a potential inhibition mechanism by which GSH-AuNC perturbs the TrxR-Trx electron-transfer pathway in *M. tuberculosis*, contributing to the future design of TrxR-targeted gold nanomaterials.

## 4. Materials and Methods

### 4.1. Synthesis and Characterization of the Gold Nanocluster

GSH-AuNC was synthesized and characterized according to a previously reported method [22,29]. Briefly, GSH-AuNC was synthesized by mixing aqueous solutions of GSH and hydrogen tetrachloroaurate trihydrate (HAuCl_4_·3H_2_O) at 25 °C for 10 min. The mixture was then reacted at 70 °C for 12 h with stirring, followed by incubation in the dark at room temperature for another 12 h, resulting in an orange-fluorescent solution. For purification, ethanol was added to the crude product to precipitate the GSH-AuNC, which was collected by centrifugation, washed with an ultrapure water/ethanol mixture (1:3, *v*/*v*), and vacuum-dried. The purified GSH-AuNC was redispersed in ultrapure water with NaOH and subjected to ultrafiltration (Millipore, Burlington, MA, USA) to remove small-molecule impurities. The resulting GSH-AuNC was stored in solution at 4 °C until use.

The morphology of the GSH-AuNC was characterized by high-resolution transmission electron microscopy. A drop of diluted GSH-AuNC aqueous solution was deposited onto a carbon-coated copper grid and dried at room temperature prior to observation. The size distribution of the GSH-AuNC was measured using a Malvern laser particle size analyzer at room temperature (Malvern Panalytical Ltd., Malvern, UK). The samples were appropriately diluted with ultrapure water before measurement to ensure good dispersion. The UV-vis absorption spectra of the GSH-AuNC were recorded using a UV-visible spectrophotometer (UV-1800, Shimadzu, Tokyo, Japan) at room temperature. The spectra were collected over a wavelength range of 200–1100 nm using a quartz cuvette, with ultrapure water as the blank. Fluorescence excitation and emission spectra of the GSH-AuNC were obtained using a fluorescence spectrophotometer (RF-5301, Shimadzu, Japan) at room temperature. The excitation spectrum was recorded by monitoring the emission at 591 nm, while the emission spectrum was collected under an excitation wavelength of 363 nm. Stability tests based on fluorescence spectra showed no significant changes under assay-relevant conditions.

### 4.2. Expression and Purification of TrxR

The gene encoding *M. tuberculosis* TrxR (residues 14–321, UniProt: P9WHH1) was synthesized and cloned into the pET-28a(+) vector between the *Nde*I and *Xho*I sites. This construct, pET-28a(+)-TrxR, contains an N-terminal 6 × His tag for purification and was transformed into *E. coli* BL21 (DE3) cells for protein overexpression. A small-scale overnight culture was inoculated into 1 L of LB medium containing kanamycin and cultivated at 180 rpm and 37 °C. When the OD_600_ reached 0.6–0.8, protein expression was induced by adding 0.2 mM IPTG, followed by overnight incubation at 150 rpm and 16 °C. Cells were harvested by centrifugation at 5000 rpm for 10 min and resuspended in buffer containing 20 mM Tris pH 8.0, 500 mM NaCl, 20 mM imidazole, 5% glycerol, and 100 μM FAD.

Cells were lysed by high-pressure homogenization, and 1 mM PMSF was added to inhibit protease activity. The crude lysate was solubilized in buffer containing 20 mM Tris pH 8.0, 20 mM imidazole, and 500 mM NaCl, followed by centrifugation at 15,000 rpm for 60 min. The supernatant was subjected to nickel affinity chromatography, with a gradient elution using 20–300 mM imidazole. TEV protease and FAD were added to the eluted sample, and the sample was dialyzed overnight at 4 °C in a buffer containing 20 mM Tris pH 7.5, 100 mM NaCl, 5 mM MgCl_2_, 2 mM *β*-mercaptoethanol, and 5% glycerol for protease cleavage. The TEV protease was subsequently removed using nickel affinity chromatography, and further purification was carried out using anion exchange chromatography (Cytiva, Marlborough, MA, USA). Homogeneous fractions were dialyzed into a buffer containing 20 mM Tris pH 7.5, 50 mM NaCl, 5 mM MgCl_2_, 1 mM TCEP, and 5% glycerol. After concentration to 30 mg/mL, the protein was rapidly frozen in liquid nitrogen and stored at −80 °C.

### 4.3. Crystallization of TrxR

Initial crystallization screening of TrxR (10 mg/mL) was performed with commercial kits (Qiagen, Germantown, MD, USA; Hampton Research, Aliso Viejo, CA, USA), using the sitting-drop vapor-diffusion method. The initial crystallization condition was 20% (*w*/*v*) PEG 3350 and 0.2 M NH_4_NO_3_. Bright yellow crystals were observed after 2–5 days and grew to maximum size within 2–4 weeks. Crystals obtained from the initial screening were crushed using a glass slide, added to 100 μL of reservoir solution, and serially diluted (1:10, 1:100, 1:1000) to prepare seeds. Crystal growth was initiated by mixing 0.8 μL of protein solution with an equal volume of reservoir solution (20% (*w*/*v*) PEG 3350, 0.2 M NH_4_NO_3_), equilibrating at 20 °C for 4–6 h, and then adding 0.2 μL of seed solution. Crystals suitable for X-ray diffraction were obtained by adding seeds diluted at a ratio of 1:100.

### 4.4. Data Collection, Processing, and Refinement

Diffraction data were collected at the BL18U1 beamline of the Shanghai Synchrotron Radiation Facility (SSRF, Shanghai, China). Data indexing, integration and scaling were performed using HKL-2000 [36]. Molecular replacement was performed using Phaser ver. 2.8 from the CCP4 suite ver. 9.0 [37], with the crystal structure of the TrxR from *M. tuberculosis* (PDB code: 2A87) [25] as the initial model. During molecular replacement calculations, the cofactor FAD was omitted from the initial model. Clear electron density corresponding to FAD was observed in both 2F_o_–F_c_ and F_o_–F_c_ difference maps at its expected binding site. Structural refinement was carried out using Phenix.refine ver. 1.16 [38] and REFMAC5 ver. 5.8 [39], and model building was performed with Coot ver. 0.9.8 [40]. Data collection and refinement statistics are summarized in Supplementary Table S1. Figures and superposition were generated using PyMOL ver. 2.6 (Schrödinger, New York, NY, USA).

### 4.5. Biotinylation and Biolayer Interferometry (BLI)

Using the constructed pET-28a(+)-TrxR plasmid as a template, a DNA sequence encoding AviTag (GLNDIFEAQKIEWHE) was inserted at the C-terminus of the TrxR gene via PCR to generate the recombinant plasmid pET-28a(+)-TrxR-Avi [41]. Then the pET-28a(+)-TrxR-Avi plasmid was co-transformed with the biotin ligase (BirA) plasmid into *E. coli* BL21(DE3) for protein overexpression. A small-scale culture was inoculated into 1 L of LB medium containing kanamycin and ampicillin, and cultured at 37 °C and at 180 rpm until the OD_600_ reached 0.6–0.8. Protein expression was then induced overnight at 16 °C and at 150 rpm by adding 0.2 mM IPTG and 50 μM biotin to achieve biotinylation modification.

The binding between GSH-AuNC and TrxR was monitored using BLI experiments on an Octet Red96 instrument (Sartorius, Goettingen, Germany). Streptavidin (SA) sensors were equilibrated in a running buffer containing 20 mM Tris pH 7.5, 150 mM NaCl, and 0.05% (*v*/*v*) Tween 20. Biotinylated TrxR (50 μg/mL) was loaded onto the sensors, achieving a loading shift of approximately 5 nm via biotin-streptavidin interaction. After reaching a stable baseline for 60–120 s, the sensors were immersed in a series of GSH-AuNC dilutions (ranging from 21–340 μM in a two-fold serial dilution) for a 360 s association phase. The dissociation phase was monitored for 360 s by immersing the sensors in buffer. A reference sensor and zero concentration (only for the control buffer) were used for double reference subtraction. The binding kinetic parameters of the interaction between GSH-AuNC and TrxR were obtained using a 1:1 binding model with global fitting via the built-in Data Analysis software.

### 4.6. Enzyme Activity Assay

The GSH-AuNC was subjected to two-fold serial dilution (a total of 8 gradients), and then each dilution was mixed with the TrxR solution and incubated at 37 °C for 10–60 min. The reaction was carried out in assay buffer containing 20 mM Tris pH 7.5 and 150 mM NaCl. The reaction was initiated by the sequential addition of NADPH, DTNB, and finally TrxR or TrxR-GSH-AuNC to reach a total volume of 200 μL. The final concentrations of TrxR, NADPH, and DTNB in the reaction system were 1 μM, 1 mM, and 1 mM, respectively, with the highest final concentration of GSH-AuNC being 35 μM. The conversion of DTNB to TNB was monitored by continuously measuring the absorbance at 412 nm for 10 min at 37 °C using a microplate reader. IC_50_ values were determined by nonlinear regression using R ver. 4.5.1 [42].

### 4.7. Cell Culture and Cytotoxicity Assay

16HBE cells were cultured in Roswell Park Memorial Institute 1640 (RPMI-1640, Buffalo, NY, USA) medium with 10% fetal bovine serum (FBS) and 1% penicillin/streptomycin (P/S). Cells were incubated in a humidified incubator at 37 °C and 5% CO_2_.

Cytotoxicity was evaluated by Cell Counting Kit-8 system (CCK-8, Biosharp, Hefei, China). Cells were seeded into 96-well plates at a density of 5 × 10^3^ cells in 100 μL of the medium per well. After 24 h, cells were treated with different doses of GSH-AuNC (2–16 μM) for an additional 24 h. The CCK-8 reagent was added and cells were incubated for 1.5 h at 37 °C. Absorbance was measured at 450 nm with a microplate reader. The cell viability was calculated and shown as the mean ± SD of five independent experiments.

### 4.8. Molecular Dynamics Simulations

The initial geometry of GSH-AuNC was constructed in GaussView ver. 5.0 and subsequently optimized with the Gaussian 09 program using density functional theory (DFT) [30]. During the DFT optimization, gold atoms were treated with the LANL2DZ basis set, whereas the 6-31G(d) basis set was assigned to carbon, hydrogen, oxygen, nitrogen, and sulfur atoms. After geometry optimization of GSH-AuNC, CHARMM-format force-field parameters were generated, with partial charges derived using the RESP fitting approach. First, based on the Au-Au bond length (3.2 Å), we determined which gold atoms in GSH-AuNC could form bonds and obtained information such as bond angles and dihedral angles. Then, we used Automated Topology Builder (ATB) and Repository (https://atb.uq.edu.au/index.py, accessed on 2 January 2026) [43] to obtain the force field description of GSH. Next, we combined gold atoms and GSH to form a complete GSH-AuNC. Finally, we used the GoIP-CHARMM [44] to provide Lennard-Jones parameters for Au-Au, Au-S, and other interactions, and combined this with the RESP charge fitting method to construct the force field parameters of the cluster.

GSiteScorer (https://proteins.plus/, accessed on 2 January 2026) [45] employs a grid-based approach, utilizing the three-dimensional structure of macromolecules and a difference-of-Gaussian filter to detect potential binding pockets and segment them into sub-pockets. This method was used to analyze the surface of protein structures and identified a total of 22 sub-binding pockets. We used the AutoDock ver. 4.2.0 software package to obtain the preferred conformation of GSH binding to proteins. Based on the GSH binding sites, 54 initial poses were prepared in Visual Molecular Dynamics (VMD) software [46], which was also used to create the necessary input files for MD simulations. Molecular dynamics was used to screen for the complex conformations that exhibited the most stable binding affinity to the protein.

All MD simulations were performed in the NPT ensemble with full periodic boundary conditions. The Langevin thermostat and the Nosé–Hoover Langevin piston algorithm were applied to maintain constant temperature and pressure, allowing the system to reach equilibrium under physiological-like conditions. Electrostatics were handled using the particle mesh Ewald (PME) method with conducting boundaries, and a cutoff distance of 12 Å was set for van der Waals interactions. The CHARMM22 and CHARMM36 force fields [47] were used to model the molecular system, with water molecules represented explicitly by the TIP3P model. Production simulations were conducted using NAMD ver. 2.13 [48], and the resulting trajectories were analyzed with VMD ver. 1.9.3. The Gibbs binding free energy was estimated using the MM/PBSA module in the CHARMM program [47], the solvation energy was calculated using the APBS ver. 3.0 program, and the statistical analysis was based on a custom Python script (version 3.11.4).

Before the production phase, the system underwent an initial energy minimization involving 5000 steepest descent steps. This was followed by a 100 ns equilibration period, during which the temperature was progressively raised to 300 K. Thermal equilibration was further supported by coupling the system to a sodium ion solution.

### 4.9. Sequence and Structural Analysis

Multiple sequence alignment of TrxR amino acid sequences from *M. tuberculosis* (UniProt ID: P9WHH1), *M. smegmatis* (UniProt ID: O30973), *E. coli* (UniProt ID: P0A9P4), *H. influenzae* (UniProt ID: P43788), *H. pylori* (UniProt ID: P56431) and *Homo sapiens* (*H. sapiens*) (UniProt ID: Q16881) was performed using CLUSTALW (https://www.genome.jp/tools-bin/clustalw, accessed on 26 August 2025) [49], and the alignment results were visualized with ESPript 3.0 (https://espript.ibcp.fr/ESPript/ESPript/index.php, accessed on 26 August 2025). Crystal structures from *M. tuberculosis* (PDB code: 2A87), *M. smegmatis* (PDB code: 8CCI), *E. coli* (PDB code: 1TDE), *H. influenzae* (PDB code: 5U63), *H. pylori* (PDB code: 2Q0L) and *H. sapiens* (PDB code: 7X1R) [18,25,26,27,28] were superimposed using PyMOL ver. 2.6.

### 4.10. AlphaFold3 Prediction

AlphaFold3 (https://alphafoldserver.com/, accessed on 12 August 2025) was used to predict the TrxR-Trx complex structure from *M. tuberculosis* [50]. Cofactors FAD and NADPH were incorporated into the prediction process to model the cofactor-bound state. The amino acid sequences of TrxR and Trx used for prediction were retrieved from UniProt (https://www.uniprot.org/, accessed on 12 August 2025) with UniProt IDs P9WHH1 and P9WG67, respectively.

## Data Availability

The data presented in this study are openly available in PDB at https://doi.org/10.2210/pdb9xub/pdb, reference number 9XUB.

## Supplementary Materials

The following supporting information can be downloaded at: https://www.mdpi.com/article/10.3390/ijms27031209/s1.
