# Supplementary material for: Structural and Mechanistic Characterization of Mycobacterium tuberculosis TrxR Inhibition by Glutathione-Coated Gold Nanocluster
Source: Int J Mol Sci. 2026 Jan 25;27(3):1209. doi: 10.3390/ijms27031209 (PMC12898023; doi:10.3390/ijms27031209)
Supplement: Supplementary file 1 [file ijms-27-01209-s001.zip › ijms-4088103-supplementary.pdf]

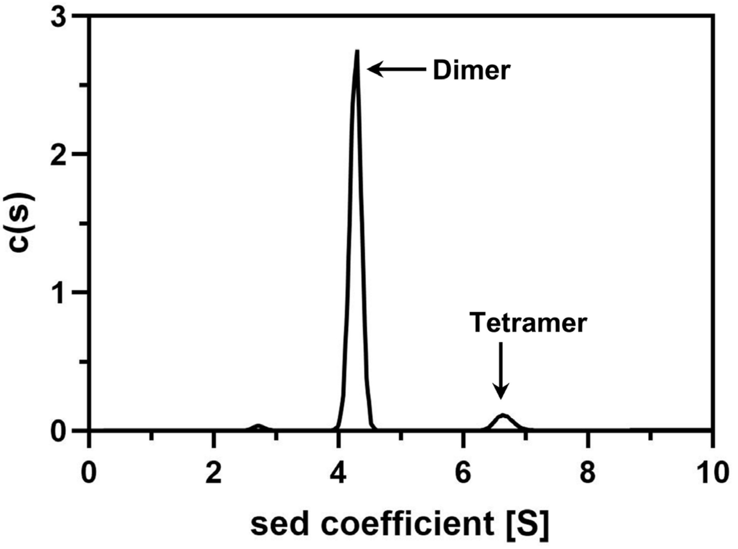


**Figure S1.** Sedimentation coefficient distribution derived from analytical ultracentrifugation data.

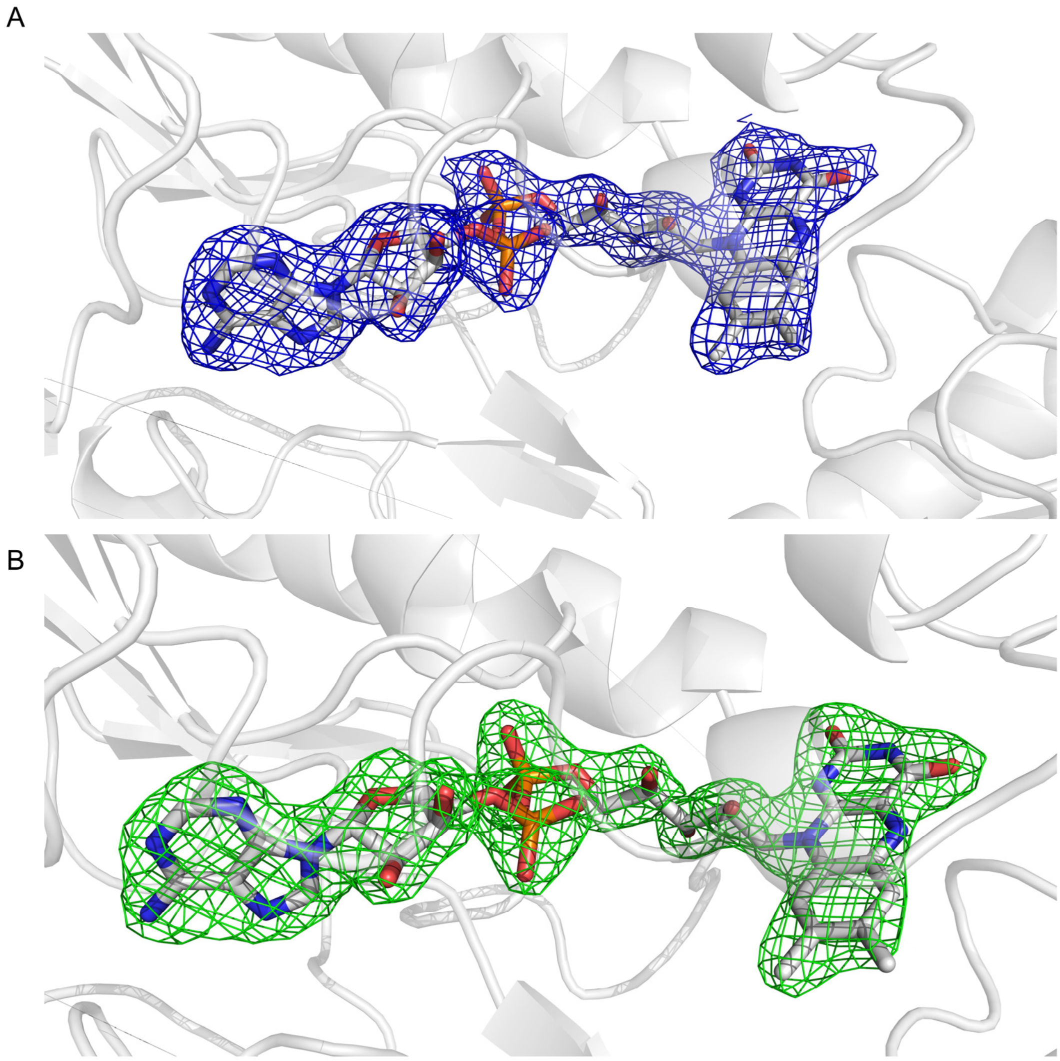


**Figure S2.** Electron density maps of the FAD cofactor of *M. tuberculosis* TrxR. **(A)** The $2F_o$-$F_c$ electron density map (contoured at 1 σ). **(B)** The $F_o$-$F_c$ omit map (contoured at 3 σ).

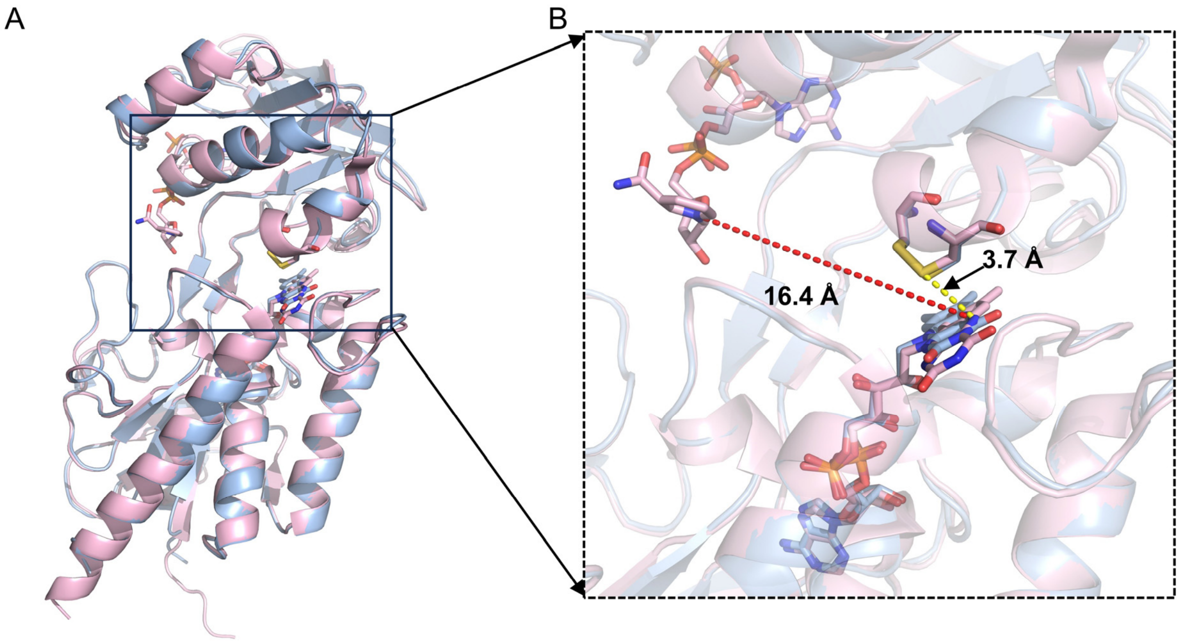


**Figure S3.** Structural comparison of *M. tuberculosis* TrxR. **(A)** Superposition of the TrxR monomer determined in this study with a previously reported *M. tuberculosis* TrxR structure (PDB code: 2A87). **(B)** Enlarged view of (A). The NADPH-FAD and FAD-disulfide distances are shown.

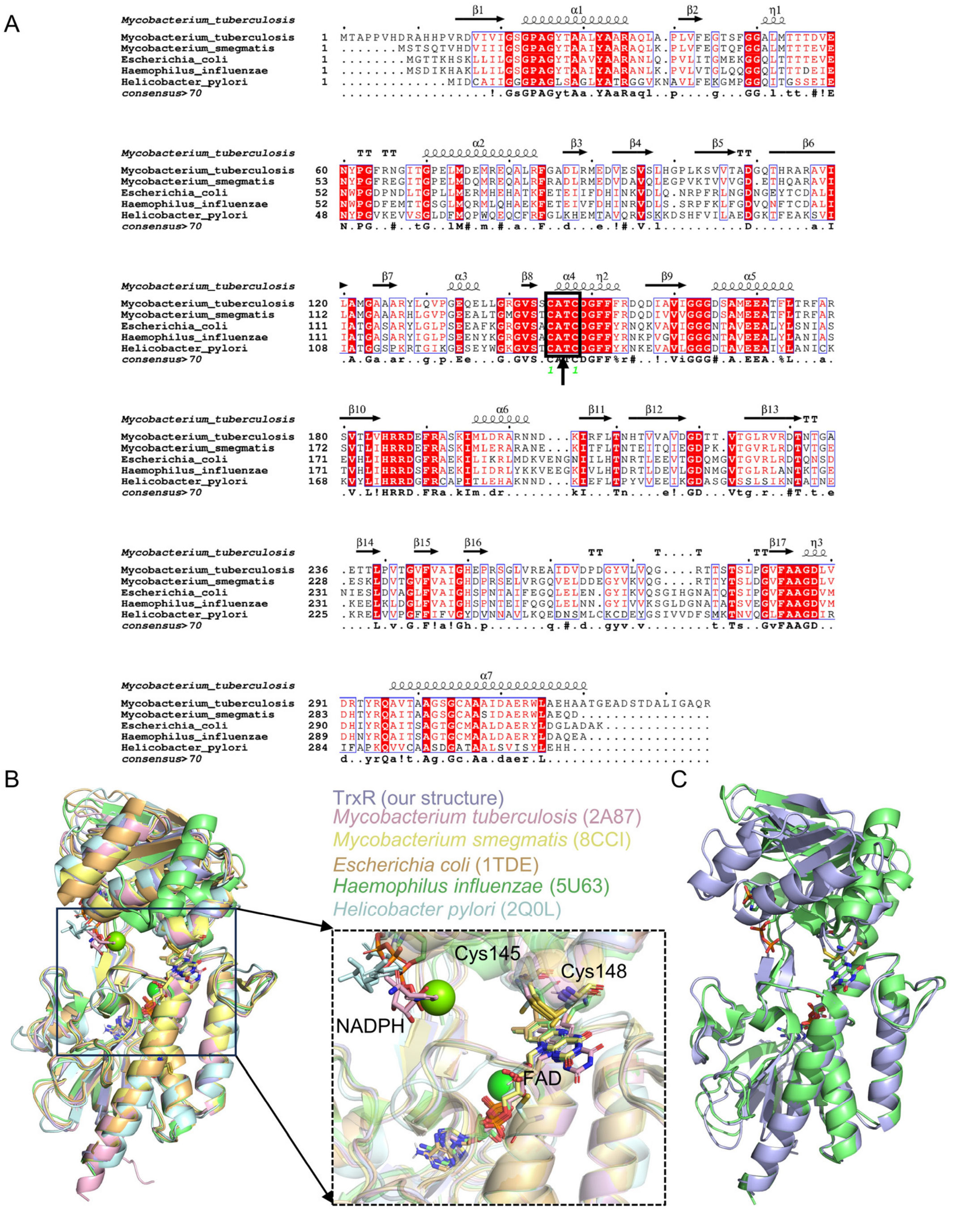


**Figure S4.** Sequence and structural alignment of TrxR monomers from different species. **(A)** Multiple sequence alignment of TrxRs from *M. tuberculosis*, *M. smegmatis*, *E. coli*, *H. influenzae*, and *H. pylori*. The conserved CXXC active-site motif in the NADPH-binding domain is indicated by a black box and an arrow. The secondary-structure elements of *M. tuberculosis* TrxR are shown above the alignment. **(B)** Structural superposition of TrxRs from *M. tuberculosis* (this study and PDB code: 2A87), *M. smegmatis* (PDB code: 8CCI), *E. coli* (PDB code: 1TDE), *H. influenzae* (PDB code: 5U63), and *H. pylori* (PDB code: 2Q0L). **(C)** Structural superposition of the FAD-binding domains from *M. tuberculosis* and *H. influenzae*.

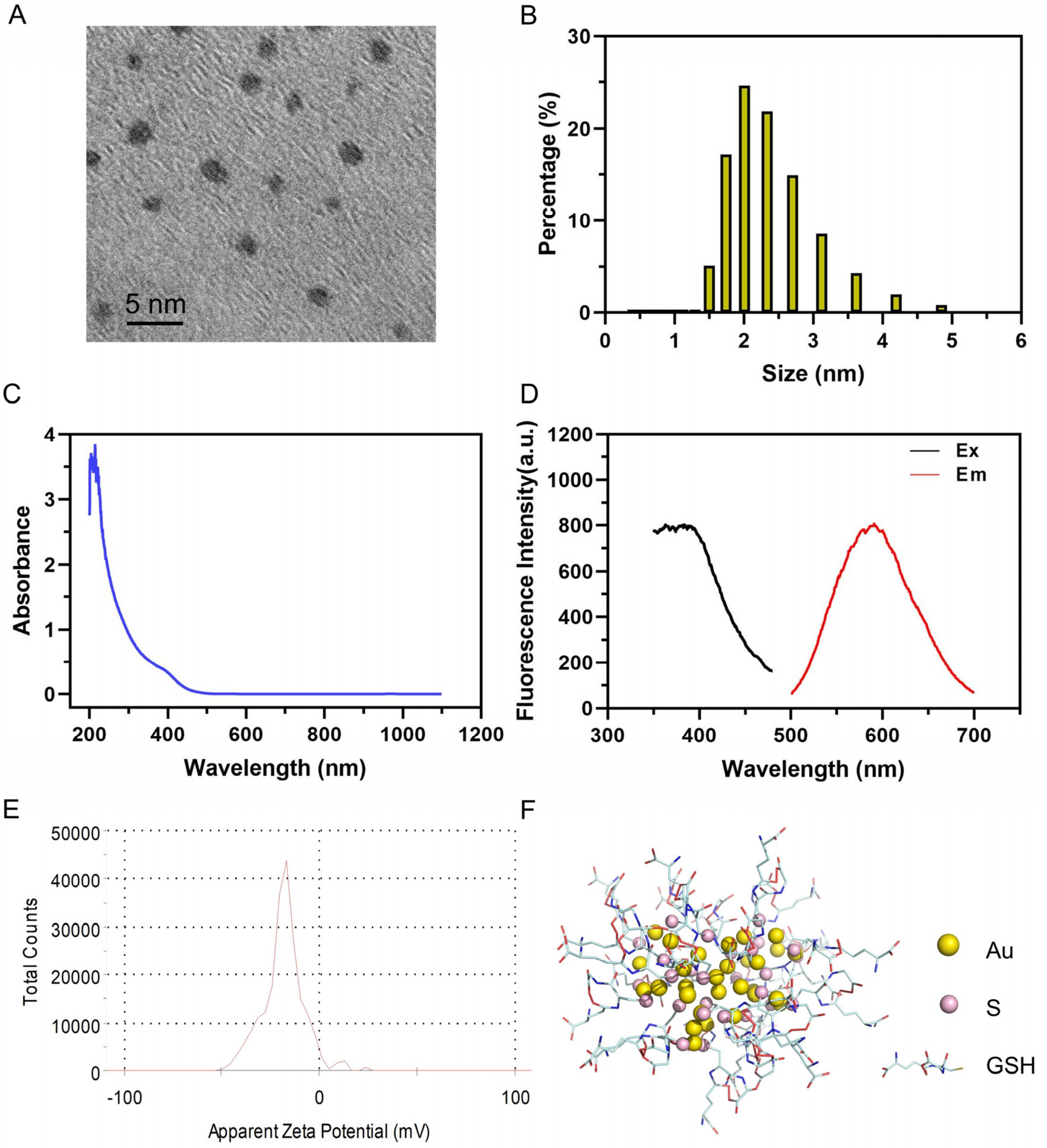


**Figure S5.** Characterization of GSH-AuNC. **(A)** High-resolution transmission electron microscope image of the GSH-AuNC. **(B)** Size distribution of the GSH-AuNC by dynamic light scattering. **(C)** UV-vis absorption (blue line) of the GSH-AuNC at room temperature. **(D)** Fluorescence excitation (black lines, λex = 363 nm) and fluorescence emission (red lines, λem = 591 nm) spectra of the GSH-AuNC at room temperature. **(E)** Zeta potential distribution of the GSH-AuNC. The result shows an average zeta potential of approximately -18.3 mV. **(F)** The molecular structure of GSH-AuNC.

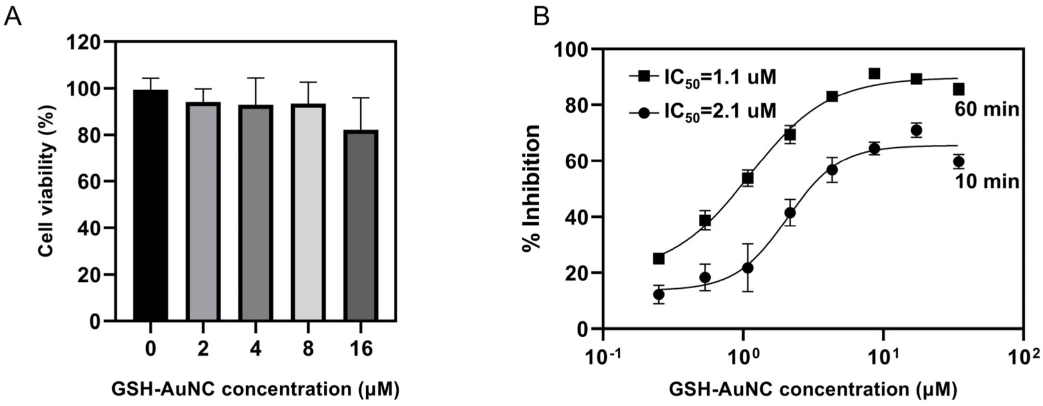


**Figure S6.** Cytotoxicity of GSH-AuNC in 16HBE cells and its inhibitory effect on TrxR activity. **(A)** 16HBE cells were treated with different concentrations of GSH-AuNC for 24 h, and the cell viability was quantified by CCK-8 assay. **(B)** Inhibition of TrxR activity by GSH-AuNC. After pre-incubation for 10 and 60 min, the $IC_{50}$ was determined to be 2.1 μM with a 95% confidence interval of 1.6-2.6 μM and 1.1 μM with a 95% confidence interval of 0.8-1.4 μM. The goodness of fit of the model was evaluated by $R^2$ values of 0.950 and 0.980, respectively. Data are presented as mean ± SD from three biological replicates.

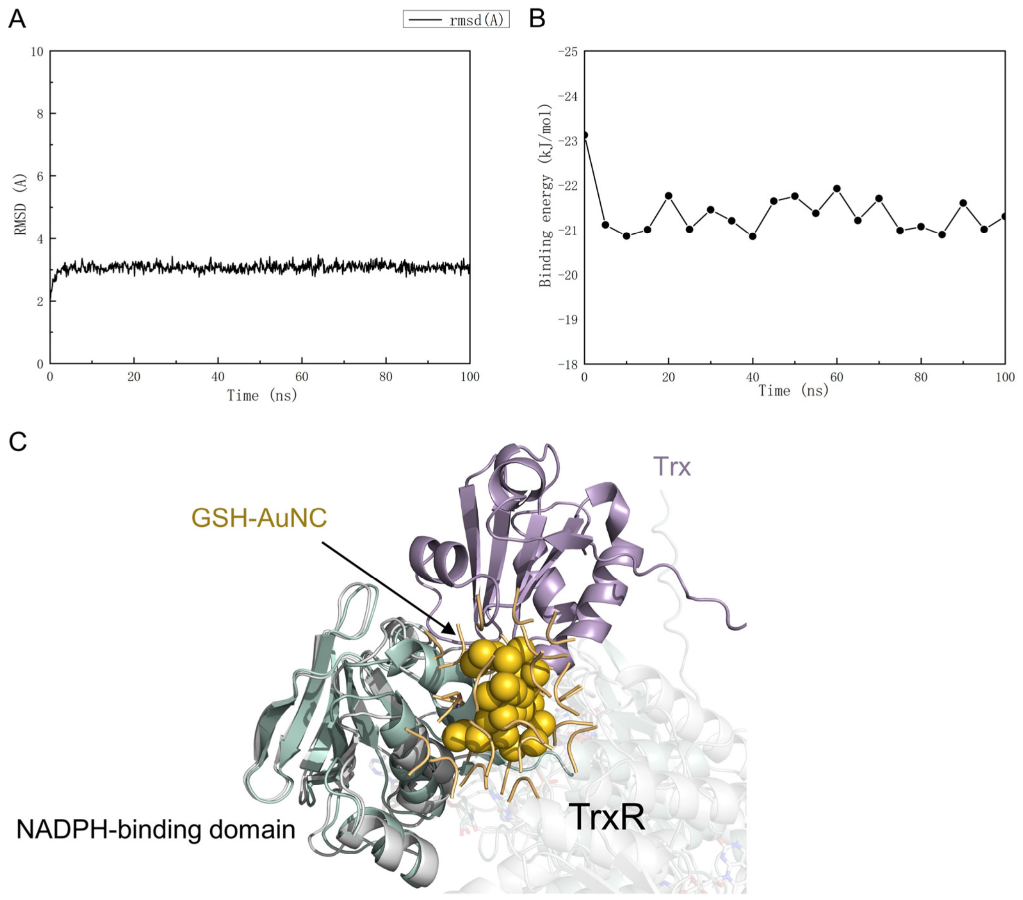


**Figure S7.** MD simulation analysis of GSH-AuNC and TrxR interaction. **(A)** The time-dependent RMSD of the GSH-AuNC-TrxR complex. **(B)** The Gibbs free binding energy between GSH-AuNC and TrxR. **(C)** Superposition of the GSH-AuNC-TrxR (GSH-AuNC and TrxR are colored orange and gray) with the AlphaFold3-predicted TrxR-Trx complex structure (TrxR and Trx are colored green and purple).

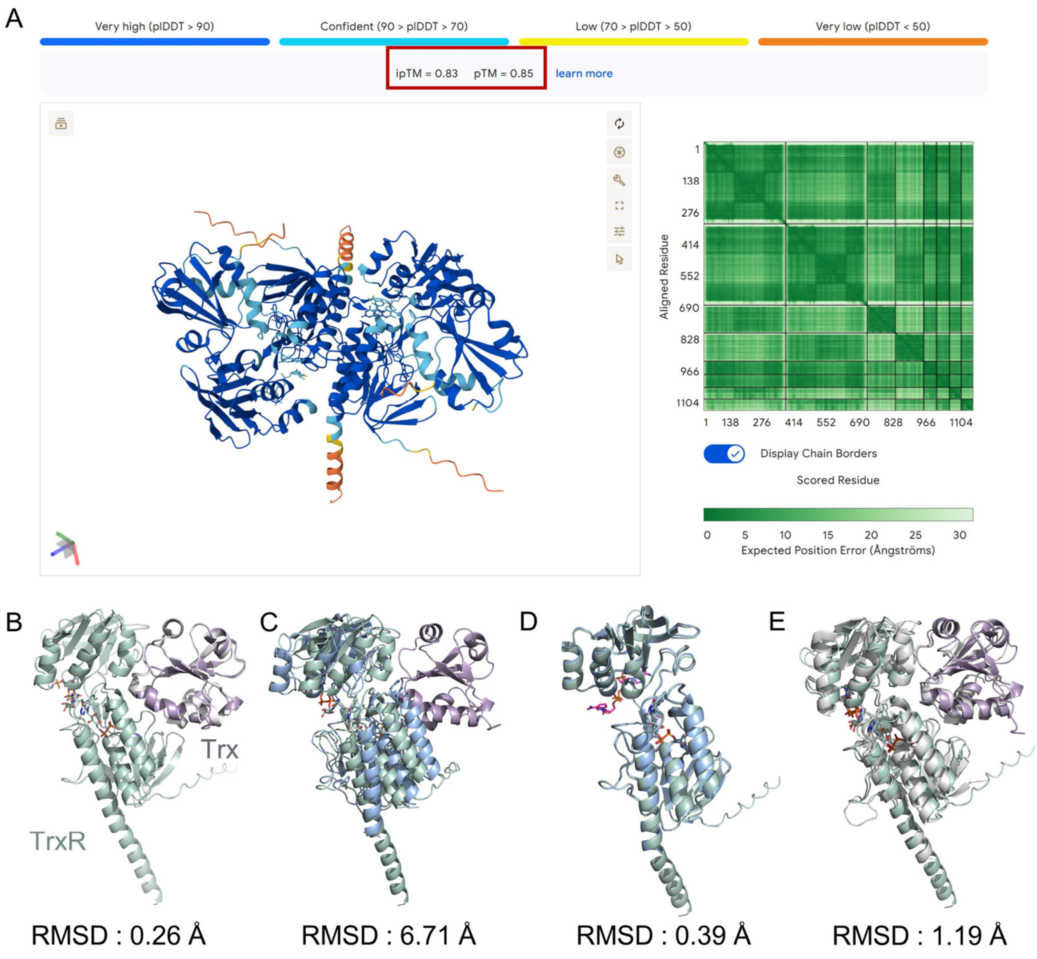


**Figure S8.** AlphaFold3 modeling results of the *M. tuberculosis* TrxR-Trx complex. **(A)** The confidence of the AlphaFold3 prediction for the TrxR-Trx complex in *M. tuberculosis*. The per-residue confidence is color-coded according to pLDDT: very high (> 90), confident (70-90), low (50-70), and very low (< 50). The predicted global scores are pTM = 0.85 and ipTM = 0.83. **(B)** Superposition of the predicted complex with the Trx crystal structure (PDB code: 2I1U), aligned on Trx. The experimental Trx is colored in gray. In the predicted model, TrxR and Trx are colored green and purple, respectively. **(C)** Superposition of the predicted TrxR-Trx complex model with the TrxR structure determined in this study, aligned on TrxR monomer. The experimental TrxR is colored in blue. **(D)** Superposition of the AlphaFold3-predicted TrxR monomer (TrxR-only prediction; in green) with the TrxR structure determined in this study (in blue). **(E)** Superposition of the predicted TrxR-Trx model with the *E. coli* TrxR-Trx structure (in gray; PDB code: 1F6M).

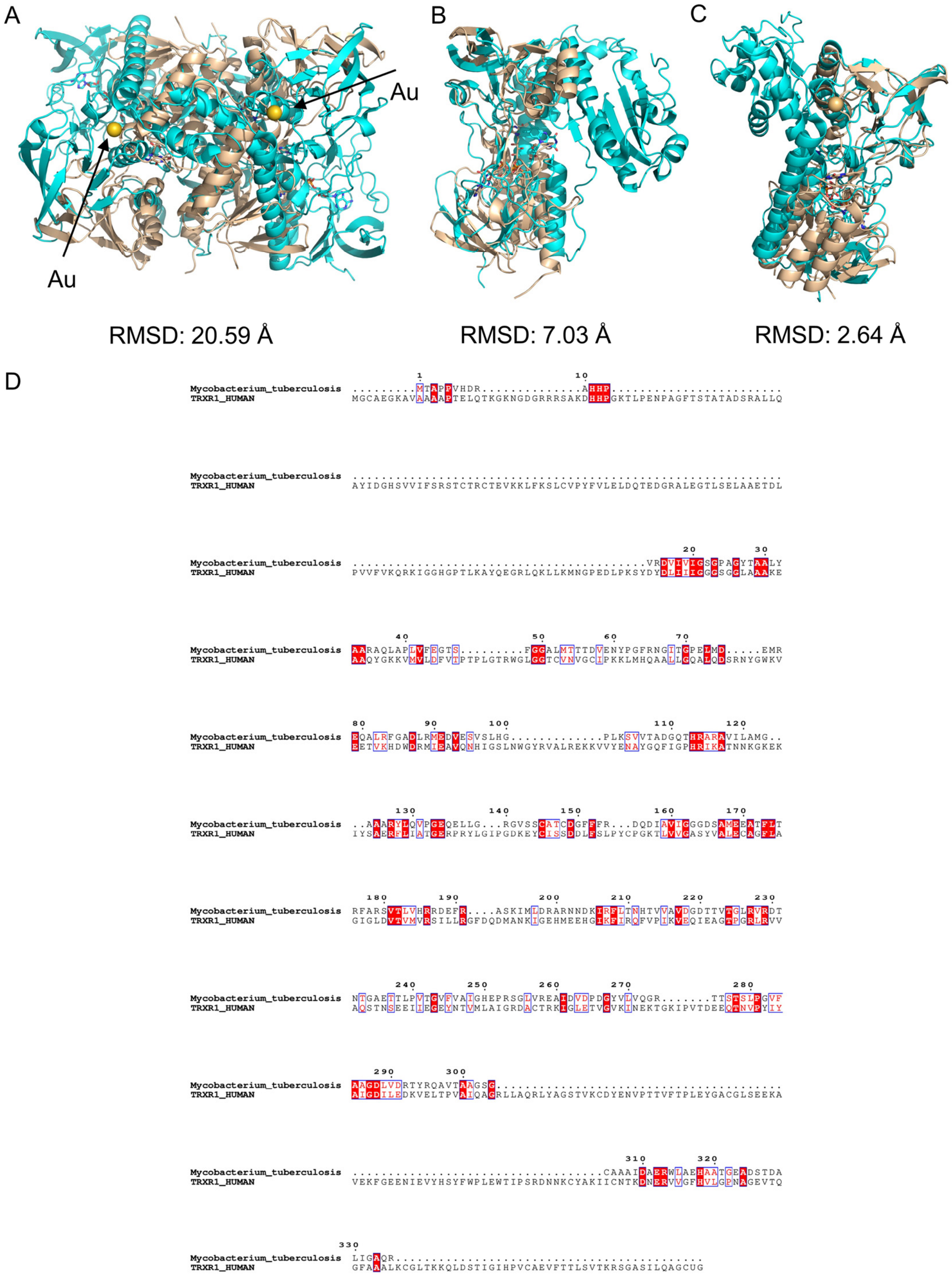


**Figure S9.** Sequence and structural alignment of hTrxR and *M. tuberculosis* TrxR. **(A)** Structure superposition of the hTrxR dimer (colored in cyan) with the *M. tuberculosis* TrxR dimer (colored in wheat). **(B)** Structure superposition of the hTrxR monomer (Cryo-EM structure of Au(I)-hTrxR complex; PDB code: 7X1R) with the *M. tuberculosis* TrxR. **(C)** Structure superposition of the hTrxR monomer with the NADPH-binding domain of *M. tuberculosis* TrxR. **(D)** Sequence alignment between hTrxR (UniProt ID: Q16881) and *M. tuberculosis* TrxR (UniProt ID: P9WHH1).

**Table S1.** Statistics for data collection and refinement.

| | *M. tuberculosis* **TrxR** |
|---|---|
| **Data collection** | |
| Diffraction source | SSRF-BL18U1 |
| Detector | Pilatus3 S 6M |
| Wavelength (Å) | 0.98 |
| Space group | $C222_1$ |
| **Cell dimensions** | |
| *a, b, c* (Å) | 99.4, 107.6, 220.4 |
| *α, β, γ* (°) | 90, 90, 90 |
| Resolution (Å) | 50-2.6 (2.69-2.60) [a] |
| $R_{merge}$ | 0.118 (0.425) |
| $R_{pim}$ | 0.032 (0.115) |
| $I/\sigma I$ | 21.3 (7.6) |
| Molecules per ASU | 4 |
| Completeness (%) | 99.9 (99.8) |
| Redundancy | 12.8 (13.1) |
| B-factor from Wilson plot (Å$^2$) | 26.5 |
| **Refinement** | |
| Resolution (Å) | 50-2.6 |
| $R_{work}/R_{free}$ | 0.180/0.232 |
| No. reflections | 36,652 |
| **No. atoms** | |
| Protein | 8448 |
| Ligand/ion | 212 |
| Water | 457 |
| B-factor | 38.1 |
| **R.m.s. deviations** | |
| Bond lengths (Å) | 0.007 |
| Bond angles (°) | 0.920 |
| **Poor rotamers (%)** | 0 |
| **Ramachandran plot** | |
| Favored (%) | 96.6 |
| Allowed (%) | 3.4 |
| Disallowed (%) | 0 |
| **PDB code** | 9XUB |

[a] The values in parenthesis mean those of the highest resolution shell.

**Table S2**. Global $C_\alpha$ RMSD values between the TrxR monomer determined in this study and homologous TrxR monomers from other species.

| PDB code | Organism | Global $C_\alpha$ RMSD (Å) |
|---|---|---|
| 2A87 | *Mycobacterium tuberculosis* | 0.38 |
| 8CCI | *Mycobacterium smegmatis* | 0.67 |
| 1TDE | *Escherichia coli* | 1.01 |
| 5U63 | *Haemophilus influenzae* | 6.83 |
| 2Q0L | *Helicobacter pylori* | 1.30 |
| 7X1R | *Homo sapiens* | 7.03 |
